# Surgical cytoreduction versus systemic therapy in patients with metastatic gastroenteropancreatic neuroendocrine neoplasms (GEP-NENS): a national cancer database analysis (NCDB)

**DOI:** 10.3389/or.2025.1589775

**Published:** 2026-01-14

**Authors:** Amr Mohamed, Fasih A. Ahmed, Omkar Pawar, Trisha Lal, Jordan Winter, John Ammori, Jeffrey Hardacre, Amit Mahipal, David Bajor, Sakti Chakrabarti, Sylvia Asa, Eva Selfridge, Madison Conces, Melissa Lumish, Sree Tirumani, Lauren Henke, Richard Hoehn

**Affiliations:** 1 Department of Medicine, Division of Hematology and Medical Oncology, University Hospitals, Seidman Cancer Center, Case Western Reserve University, Cleveland, OH, United States; 2 Department of Surgical Oncology, University Hospitals, Seidman Cancer Center, Case Western Reserve University, Cleveland, OH, United States; 3 Department of Surgery, Stanford University School of Medicine, Palo Alto, CA, United States; 4 Department of Pathology, University Hospitals, Seidman Cancer Center, Case Western Reserve University, Cleveland, OH, United States; 5 Department of Radiology, University Hospitals, Seidman Cancer Center, Case Western Reserve University, Cleveland, OH, United States; 6 Department of Radiation Oncology, University Hospitals, Seidman Cancer Center, Case Western Reserve University, Cleveland, OH, United States

**Keywords:** gastroenteropancreatic, neuroendocrine, neoplasms, surgical cytoreduction, systemic therapy

## Abstract

**Background:**

The optimal role of surgical cytoreduction in metastatic gastroenteropancreatic neuroendocrine neoplasms (GEP-NENs) remains uncertain, as supporting evidence is largely retrospective and rarely compares surgery with contemporary systemic therapy. Using a national cancer registry, we evaluated overall survival (OS) associated with cytoreductive surgery compared with systemic therapy alone.

**Methods:**

Adult patients with stage IV well-differentiated GEP-NENs were identified in the National Cancer Database (2004–2020). Demographic, tumor, and facility variables stratified patients. Treatment groups included cytoreductive surgery (CRS) alone, CRS plus systemic chemotherapy, and systemic therapy alone. Overall survival (OS) was compared using Kaplan-Meier (KM) analysis and multivariable Cox proportional hazards models.

**Results:**

Among 3,183 patients with stage IV GEP-NENs, 69.8% underwent cytoreductive surgery (CRS) alone, 6.7% received CRS plus systemic chemotherapy, and 23.4% received systemic therapy alone. Median overall survival (OS) differed significantly by treatment: CRS alone, 140.9 months; CRS plus chemotherapy, 96.2 months; and systemic therapy alone, 51.6 months (p < 0.001). The survival advantage of CRS persisted across histologic grades, including both G1–G2 tumors (140.9 vs. 96.2 vs. 53.6 months, p < 0.001) and G3 well-differentiated tumors (39.8 vs. 13.1 vs. 9.6 months, p < 0.001). Survival benefits were also observed across primary tumor sites. In midgut NENs, median OS was 157.6 vs. 99.2 vs. 87.5 months (p < 0.001), and in pancreatic NENs, 117.5 months vs. not reached vs. 50.8 months (p < 0.001). On multivariable analysis, older age, lower SES, higher comorbidity burden, colon or rectal primaries, positive margins, and higher tumor grade were associated with worse survival. Longer time from diagnosis to surgery (>35 days) was associated with improved survival. CRS remained independently associated with improved OS (HR 0.80, 95% CI 0.67-0.94), while receipt of systemic chemotherapy was associated with increased mortality (HR 1.71, 95% CI 1.36-2.17).

**Conclusion:**

Surgical cytoreduction was associated with significantly improved survival compared with systemic therapy alone in metastatic GEP-NENs, with consistent benefits across histologic grades and primary tumor sites. These findings support considering CRS in appropriately selected patients and underscore the need for prospective validation.

## Introduction

Neuroendocrine neoplasms (NENs) are a heterogeneous group of neoplasms that arise from endocrine cells in numerous organs, with over 60% occurring in the gastrointestinal tract (1, 2). Their incidence has increased substantially over the past several decades, driven in part by advances in diagnostic imaging and pathology. Nearly half of patients present with metastatic disease, most commonly to the liver, where hepatic involvement is a significant determinant of morbidity and the leading cause of disease-specific mortality.

The World Health Organization (WHO) classification of NENs has undergone important refinements over the past decade. The 2010 classification distinguished well-differentiated NENs from poorly differentiated neuroendocrine carcinomas (NECs) but classified all tumors with a Ki-67 index >20% as NEC (3). Subsequent updates in both the 2017 and 2019 classifications introduced a novel category: well-differentiated G3 NENs, defined by tumor morphology despite a high proliferation index (Ki-67 > 20%). These tumors are biologically and clinically distinct from poorly differentiated NEC and now represent a recognized subgroup within the NEN spectrum (4–7). Management of metastatic gastropancreatic-NENs (GEP-NENs) often requires a multidisciplinary approach that includes systemic therapy, liver-directed treatments, and cytoreductive surgery (CRS). Current international guidelines recommend systemic therapy as first-line treatment for metastatic NENs, with chemotherapy primarily used for higher-grade or rapidly progressive tumors (8). Previous retrospective studies and single institutions’ experience suggest that CRS of liver metastases may alleviate symptoms and improve overall survival (9–15). However, these procedures can be associated with substantial morbidity and mortality. While the optimal extent of CRS remains debated, prior studies suggest that substantial debulking may confer symptomatic and survival benefits (11, 12).

Despite these observations, most prior studies compared surgical outcomes with historical controls rather than contemporary systemic therapy in metastatic GEP-NENs. As a result, the relative benefit of surgical *versus* medical cytoreduction remains uncertain. To address this gap, we performed a comparative analysis of patients with metastatic GEP-NENs in the National Cancer Database (NCDB) to evaluate overall survival differences among CRS, systemic chemotherapy, and their combination.

## Methods

### Data source and study population

The NCDB is a large, nationally recognized cancer registry that represents more than 70% of all newly diagnosed cancers in the US. We identified adults aged 18 or older diagnosed between 2004 and 2020 with stage IV GEP-NENs. Primary tumors were included using ICD-10 codes for the pancreas (C250-4, C257-9), stomach (C160-6, C168-9), small intestine (C170-173, C178-9), appendix (C181), colon (C180, C182-9), rectum (C209), and intestines not otherwise specified (C260). Neuroendocrine histology was confirmed using ICD-O-3 morphology codes 8150-3, 8150–8156, 8155-6, 8240, 8243-8246, 8013, 8574, and 8249 (16).

### Tumor grade classification

Because histologic grading conventions changed during the study period, we constructed a composite variable to ensure consistent organization. For patients diagnosed between 2004 and 2018, ICD-O-3 grades I and II were classified as G1 (well-differentiated) and G2 (moderately differentiated), respectively. For cases diagnosed in 2018 and later, the WHO 5^th^ edition classifications were used to identify G3 well-differentiated tumors. Poorly differentiated NECs from both histologic classifications were excluded from the study.

### Exclusion criteria

Patients were excluded if they had missing data on receipt of CRS or systemic chemotherapy; missing or ambiguous information on primary tumor site, metastatic site, or key demographic variables; histology consistent with poorly differentiated NECs; or missing overall survival or follow-up information.

### Treatment definitions

Cytoreductive surgery was defined using codes 2, 4, and 5 (non-primary surgical procedure to other regional sites, non-primary surgical procedure to other distal sites, or a combination of the two) of the variable for surgical procedure to other sites. Systemic therapy was defined using code 1-3 (chemotherapy administered as first-line therapy) of the variable RX_SUMM_CHEMO. Patients were categorized into three groups: CRS alone, CRS plus systemic chemotherapy, and systemic chemotherapy alone.

### Covariates

We examined a comprehensive set of demographics, clinical, and facility-level covariates. Demographic variables included age group, sex, race and ethnicity, insurance status, and socioeconomic status (SES). A 7-point composite score incorporating education and income variables was used as a proxy for SES (17). The score is arranged in ascending order, with seven corresponding to the highest SES. Clinical characteristics included the Charlson-Deyo comorbidity score (0, 1, ≥2), primary tumor site, tumor grade, presence of lymphovascular invasion, surgical margin status, metastatic pattern (hepatic-only vs. extrahepatic disease), and days from diagnosis to surgery, dichotomized at the cohort median. Facility-level variables included facility type (Academic/Research, Community, Comprehensive Community, or Integrated Network Cancer Programs), and facility volume, categorized as low (<96 cases), medium (96-416 cases), or high (>416 cases) based on total NEN case volume. Community designation (metro, urban, or rural) and distance traveled for care (<96.7 vs. ≥96.7 miles, using the cohort median) were also considered.

### Outcomes and statistical analysis

The primary outcome was overall survival (OS), estimated using the Kaplan-Meier (KM) method and compared using log-rank testing. Multivariable Cox proportional hazards models were constructed to evaluate independent predictors of OS, adjusting for age, sex, race/ethnicity, SES index, Charlson-Deyo comorbidity score, facility type and volume, community designation, distance traveled, primary tumor site, days from diagnosis to surgery, surgical margins, lymphovascular invasion, tumor grade, and receipt of CRS or systemic therapy.

A sensitivity analysis was performed, restricting the cohort to G1 and G2 well- and moderately differentiated tumors to assess the robustness of the findings. A separate G3-only analysis was not feasible due to the small sample size. All analyses were conducted using a two-sided significance threshold of p < 0.05.

This study was exempt from institutional review board oversight because the NCDB data are de-identified.

## Results

### Baseline clinical and demographic characteristics

Baseline clinicodemographic characteristics are shown in Table 1. Among the 3,183 patients, 2,222 (69.8%) underwent CRS alone, 214 (6.7%) received CRS plus systemic chemotherapy, and 747 (23.4%) received systemic chemotherapy alone. Patients receiving CRS alone were slightly older, with 32.8% aged 60–69 years, whereas combined-modality patients had the highest proportion under age 50 (26.2%). Sex distribution was similar across groups, though the systemic chemotherapy group had a higher proportion of males (55.6%).

**TABLE 1:** Baseline clinical and demographic characteristics.

​	Cytoreductive surgery (CRS)	CRS and systemic chemotherapy	Systemic chemotherapy	P-value
n	2,222 (69.8%)	214 (6.7%)	747 (23.4%)	​
Age				
<50	380 (17.1%)	56 (26.2%)	133 (17.8%)	<0.001
50–59	574 (25.9%)	64 (29.9%)	242 (32.4%)	​
60–69	727 (32.8%)	62 (29.0%)	217 (29.1%)	​
70–79	443 (20.0%)	30 (14.0%)	124 (16.6%)	​
80–89	94 (4.2%)	2 (0.9%)	30 (4.0%)	​
Sex				
Male	1,055 (47.5%)	104 (48.6%)	415 (55.6%)	<0.001
Female	1167 (52.5%)	110 (51.4%)	332 (44.4%)	​
Race and ethnicity				
Non-Hispanic White	1,761 (79.3%)	163 (76.2%)	549 (73.5%)	<0.001
Non-Hispanic Black	235 (10.6%)	21 (9.8%)	110 (14.7%)	​
Hispanic	129 (5.8%)	10 (4.7%)	46 (6.2%)	​
Non-Hispanic others and unknown	97 (4.4%)	20 (9.3%)	42 (5.6%)	​
SES index				
1 (lowest)	135 (7.3%)	17 (9.4%)	74 (11.7%)	0.021
2	183 (9.9%)	19 (10.6%)	73 (11.6%)	​
3	253 (13.6%)	18 (10.0%)	70 (11.1%)	​
4	268 (14.4%)	30 (16.7%)	79 (12.5%)	​
5	299 (16.1%)	33 (18.3%)	86 (13.7%)	​
6	337 (18.2%)	36 (20.0%)	120 (19.0%)	​
7 (highest)	381 (20.5%)	27 (15.0%)	128 (20.3%)	​
Insurance				
Not insured	30 (1.4%)	7 (3.3%)	18 (2.4%)	<0.001
Private insurance/Managed care	1152 (51.8%)	132 (61.7%)	395 (52.9%)	​
Medicaid	120 (5.4%)	9 (4.2%)	66 (8.8%)	​
Medicare	867 (39.0%)	59 (27.6%)	254 (34.0%)	​
Other/Unknown	53 (2.4%)	7 (3.3%)	14 (1.9%)	​
Charlson-deyo score				
0	1,624 (73.1%)	161 (75.2%)	573 (76.7%)	0.34
1	438 (19.7%)	38 (17.8%)	132 (17.7%)	​
≥2	160 (7.2%)	15 (7.0%)	42 (5.6%)	​
Facility type				
Academic/Research	1237 (58.5%)	104 (54.2%)	341 (48.4%)	<0.001
Community cancer	50 (2.4%)	5 (2.6%)	37 (5.3%)	​
Comprehensive community cancer	482 (22.8%)	53 (27.6%)	216 (30.7%)	​
Integrated network cancer	346 (16.4%)	30 (15.6%)	110 (15.6%)	​
Facility volume				
<96	267 (12.0%)	27 (12.6%)	170 (22.8%)	<0.001
96–416	981 (44.1%)	100 (46.7%)	324 (43.4%)	​
>416	974 (43.8%)	87 (40.7%)	253 (33.9%)	​
Distance traveled				
<96.7 miles	1638 (73.7%)	152 (71.0%)	584 (78.2%)	0.025
≥96.7 miles	584 (26.3%)	62 (29.0%)	163 (21.8%)	​
Community designation				
Metro	1774 (85.9%)	175 (87.1%)	611 (86.5%)	0.32
Urban	262 (12.7%)	21 (10.4%)	79 (11.2%)	​
Rural	29 (1.4%)	5 (2.5%)	16 (2.3%)	​
Primary site	​	-	​	​
Pancreas	429 (19.3%)	83 (38.8%)	390 (52.2%)	<0.001
Stomach	34 (1.5%)	7 (3.3%)	34 (4.6%)	​
Small intestine	1631 (73.4%)	106 (49.5%)	232 (31.1%)	​
Appendix	19 (0.9%)	4 (1.9%)	6 (0.8%)	​
Colon	82 (3.7%)	11 (5.1%)	46 (6.2%)	​
Rectum	27 (1.2%)	3 (1.4%)	39 (5.2%)	​
Days from diagnosis to surgery	​	​	​	0.33
≤35	1107 (49.8%)	117 (54.7%)	NA	​
>35	1115 (50.2%)	97 (45.3%)	NA	​
Surgical margins	​	​	​	​
Negative	1,603 (77.4%)	127 (70.9%)	NA	0.069
Positive	468 (22.6%)	52 (29.1%)	NA	​
Lymphovascular invasion	​	​	​	​
Yes	1,488 (79.1%)	130 (81.3%)	162 (62.0%)	<0.001
Grade				
G1/Well-differentiated	1,502 (67.7%)	137 (64.6%)	474 (64.7%)	<0.001
G2/Moderately-differentiated	700 (31.5%)	68 (32.1%)	241 (32.9%)	​
G3	17 (0.8%)	7 (3.3%)	18 (2.5%)	​
Metastatic pattern				
Hepatic-only	1477 (76.9%)	136 (73.1%)	511 (77.5%)	0.44
Extrahepatic	444 (23.1%)	50 (26.9%)	148 (22.5%)	​

Race and ethnicity differed significantly by treatment: non-Hispanic White patients comprised the majority in each group (79.3% in CRS, 76.2% in CRS plus systemic chemotherapy, 73.5% in systemic chemotherapy; p < 0.001). SES also varied, with the systemic-only group having the highest percentage of patients with the lowest SES (Index 1: 11.7%) compared with CRS (7.3%). Insurance status differed across treatment modalities (p < 0.001): most patients across groups were privately insured or on Medicare. Comorbidity burden was similar across groups (p = 0.34).

Significant differences were observed by facility type (p < 0.001): CRS was most frequently performed at academic centers. A similar pattern was observed with facility volume: CRS was performed more often at medium- or high-volume centers (p < 0.001). Travel distance also differed modestly (p = 0.025), with CRS patients more likely to travel over 96.7 miles (26.3%) than systemic-only patients (21.8%). Community designation did not differ significantly across groups.

Primary tumor site varied markedly (p < 0.001): small intestine NENs predominated in the CRS group (73.4%), where pancreatic NENs were most common in the systemic-only group (52.2%). Time from diagnosis to surgery did not differ between groups (p = 0.33). Rates of positive surgical margins were also similar across groups (p = 0.069), and lymphovascular invasion was most frequent in the CRS plus systemic chemotherapy group (81.3%) and least frequent in the systemic-only group (62.0%). Tumor grade distributions also differed (p < 0.001): most tumors were G1/well-differentiated or G2/moderately-differentiated, while G3 well-differentiated tumors were uncommon (0.8%-3.3%). Metastatic pattern did not differ significantly (p = 0.44), with hepatic-only metastases present in approximately three-quarters of patients across all treatment groups.

### Survival outcomes by treatment modality and clinical subgroups

**FIGURE 1:**
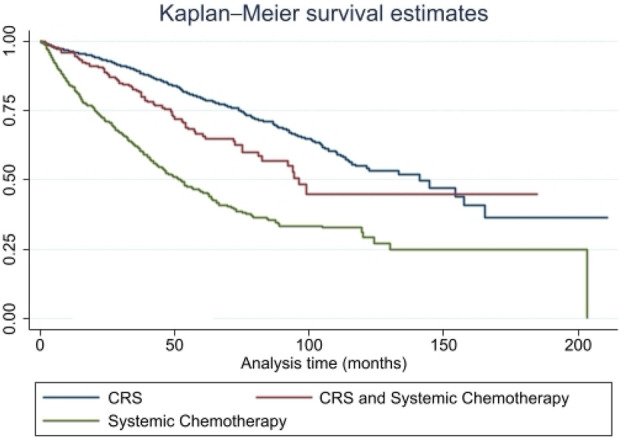
Overall Survival by Treatment Modality in Metastatic GEP-NENs. Kaplan-Meier survival curves demonstrating median overall survival (OS) among patients with metastatic gastroenteropancreatic neuroendocrine neoplasms (GEP-NENs) treated with cytoreductive surgery (CRS), CRS plus systemic chemotherapy, or systemic chemotherapy alone. CRS plus chemotherapy was associated with significantly improved OS compared with chemotherapy alone. Abbreviations: GEP-NEN, gastroenteropancreatic neuroendocrine neoplasm; CRS, cytoreductive surgery; OS, overall survival.

In univariate analysis, overall survival differed significantly by treatment modality (Figure 1). Median OS was longest among patients who underwent CRS alone (140.9 months), intermediate among those who received CRS with systemic chemotherapy (96.2 months), and shortest among those receiving systemic chemotherapy alone (51.6 months) (p < 0.001). When stratified by histological grade, this survival advantage persisted across both G1-G2 tumors (140.9 vs. 96.2 vs. 53.6 months, p < 0.001) and G3 well-differentiated tumors (39.8 vs. 13.1 vs. 9.6 months, p < 0.001).

**FIGURE 2:**
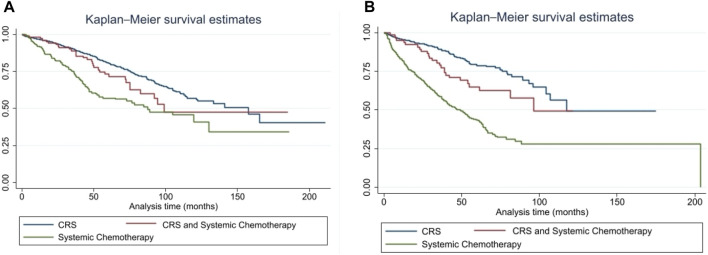
**(A)** Overall Survival by Treatment in Midgut (Small Bowel) NENs. Kaplan-Meier curves showing OS among patients with metastatic small bowel neuroendocrine neoplasms (NENs) treated with CRS, CRS plus systemic chemotherapy, or systemic chemotherapy alone. CRS plus chemotherapy was associated with significantly prolonged OS compared with chemotherapy alone. Abbreviations: NEN, neuroendocrine neoplasm; CRS, cytoreductive surgery; OS, overall survival. **(B)** Overall Survival by Treatment in Pancreatic NENs. Kaplan-Meier curves showing OS among patients with metastatic pancreatic neuroendocrine neoplasms (NENs) treated with CRS, CRS plus systemic chemotherapy, or systemic chemotherapy alone. CRS plus chemotherapy was associated with longer OS than chemotherapy alone. Abbreviations: NEN, neuroendocrine neoplasm; CRS, cytoreductive surgery; OS, overall survival.

Stratification by primary tumor site demonstrated similar patterns. Among patients with midgut small bowel NENs, CRS (with or without chemotherapy) was associated with more prolonged survival compared with systemic therapy alone (157.6 vs. 99.2 vs. 87.5 months, p < 0.001) (Figure 2A). In pancreatic NENs, cytoreduction also conferred improved survival (117.5 months vs. not reached vs. 50.8 months, p < 0.001) (Figure 2B).

### Adjusted predictors of survival

On multivariable Cox regression analysis (Table 2), several factors were independently associated with worse overall survival. Increasing age demonstrated a strong dose-response relationship: compared with patients <50 years, mortality risk was significantly higher for those aged 60-69 (HR 2.64, 95% CI 1.81-3.85), 70-79 (HR 3.72, 95% CI 2.46-5.62), and 80–89 years (HR 9.13, 95% CI 5.83-14.28) (all p < 0.001). Lower SES was also associated with worse survival, with SES index demonstrating a 49% increased hazard of death compared with the highest SES (HR 1.49, 95% CI 1.06-2.07). A higher comorbidity burden was associated with worse outcomes, with a Charlson-Deyo score of two or greater associated with a 55% increased hazard of death (HR 1.55, 95% CI 1.19-2.01, p = 0.001).

**TABLE 2:** Multivariable cox regression analysis of factors associated with overall survival.

	All tumor grades		G1/Well-differentiated and G2/Moderately-differentiated	
	HR (95% CI)	P-value	HR (95% CI)	P-value
Age (years)				
<50	Ref	​	Ref	​
50–59	1.47 (0.99–2.17)	0.053	1.50 (1.01–2.23)	0.043
60–69	2.64 (1.81–3.85)	<0.001	2.70 (1.84–3.96)	<0.001
70–79	3.72 (2.46–5.62)	<0.001	3.83 (2.51–5.83)	<0.001
80–89	9.13 (5.83–14.28)	<0.001	9.23 (5.85–14.57)	<0.001
Sex				
Male	Ref	​	Ref	​
Female	0.88 (0.75–1.04)	0.131	0.88 (0.75–1.03)	0.107
Race and ethnicity				
Non-Hispanic White	​	​	Ref	​
Non-Hispanic Black	1.18 (0.91–1.53)	0.200	1.21 (0.94–1.58)	0.142
Hispanic	0.95 (0.64–1.42)	0.811	0.96 (0.63–1.44)	0.828
Non-Hispanic others and unknown	0.90 (0.60–1.34)	0.611	0.92 (0.61–1.38)	0.679
SES index				
7 (highest)	Ref	​	Ref	​
6	1.06 (0.82–1.39)	0.648	1.07 (0.82–1.40)	0.633
5	1.20 (0.87–1.52)	0.191	1.21 (0.92–1.59)	0.171
4	1.15 (0.87–1.52)	0.340	1.12 (0.84–1.49)	0.439
3	1.05 (0.78–1.40)	0.759	1.04 (0.77–1.39)	0.809
2	1.35 (1.00–1.83)	0.053	1.34 (0.99–1.82)	0.059
1	1.49 (1.06–2.07)	0.019	1.44 (1.03–2.01)	0.034
Insurance status				
Private insurance	Ref	​	Ref	​
Not insured	0.73 (0.34–1.55)	0.411	0.71 (0.33–1.53)	0.387
Medicaid	1.17 (0.79–1.74)	0.422	1.10 (0.73–1.65)	0.660
Medicare	1.15 (0.93–1.43)	0.204	1.13 (0.91–1.41)	0.263
Other/Unknown	1.89 (1.11–3.21)	0.019	1.88 (1.11–3.21)	0.019
Charlson-deyo score				
0	Ref	​	Ref	​
1	1.05 (0.86–1.28)	0.620	1.06 (0.86–1.29)	0.590
≥2	1.55 (1.19–2.01)	0.001	1.59 (1.22–2.07)	0.001
Facility type				
Academic/Research	Ref	​	Ref	​
Community cancer	1.30 (0.85–1.98)	0.223	1.30 (0.85–1.99)	0.222
Comprehensive community cancer	1.09 (0.87–1.37)	0.471	1.07 (0.85–1.34)	0.581
Integrated network cancer	1.16 (0.91–1.48)	0.230	1.15 (0.90–1.47)	0.270
Facility volume				
<96	Ref	​	Ref	​
96–416	0.91 (0.73–1.15)	0.454	0.91 (0.72–1.14)	0.420
>416	0.86 (0.64–1.15)	0.323	0.85 (0.63–1.15)	0.289
Community designation				
Metro	Ref	​	Ref	​
Urban	0.99 (0.77–1.24)	0.867	1.00 (0.79–1.28)	0.972
Rural	1.17 (0.68–2.01)	0.576	1.18 (0.68–2.03)	0.553
Primary site				
Pancreas	Ref	​	Ref	​
Stomach	1.07 (0.56–2.03)	0.831	1.04 (0.53–2.03)	0.905
Small intestine	0.83 (0.65–1.06)	0.138	0.82 (0.64–1.05)	0.120
Appendix	1.12 (0.53–2.38)	0.761	1.10 (0.52–2.33)	0.805
Colon	1.44 (1.01–2.05)	0.045	1.36 (0.94–1.96)	0.099
Rectum	2.02 (1.26–3.23)	0.003	1.97 (1.22–3.19)	0.005
Days from diagnosis to surgery				
≤35	Ref	​	Ref	​
>35	0.79 (0.67–0.94)	0.007	0.81 (0.68–0.96)	0.017
Surgical margins				
Negative	Ref	​	Ref	​
Positive	1.51 (1.27–1.78)	<0.001	1.51 (1.28–1.80)	<0.001
Grade				
G1/Well-differentiated	Ref	​	Ref	​
G2/Moderately-differentiated	1.38 (1.16–1.64)	<0.001	1.37 (1.15–1.63)	<0.001
G3	4.34 (2.44–7.71)	<0.001	​	​
Systemic chemotherapy				
No	Ref	​	Ref	​
Yes	1.71 (1.36–2.17)	<0.001	1.77 (1.39–2.24)	<0.001
Cytoreduction				
No	Ref	​	Ref	​
Yes	0.80 (0.68–0.95)	0.011	0.80 (0.67–0.94)	0.008
Metastatic pattern				
Hepatic-only	Ref	​	Ref	​
Extrahepatic	0.99 (0.81–1.21)	0.943	0.99 (0.81–1.20)	0.910

Primary tumor site influenced outcomes, with colon (HR 1.44, 95% CI 1.01-2.05) and rectal primaries (HR 2.02, 95% CI 1.26-3.23) associated with higher mortality compared with pancreatic NENs. Delayed surgery (>35 days from diagnosis) was associated with lower mortality (HR 0.79, 95% CI 0.67-0.94, p = 0.007). Positive surgical margins were associated with significantly worse survival (HR 1.51, 95% CI 1.27-1.78, p < 0.001). Tumor grade showed a graded association with mortality: G2/moderately differentiated tumors (HR 1.38, 95% CI 1.16-1.64) and G3 well-differentiated tumors (HR 4.34, 95% CI 2.44-7.71) were both associated with poorer outcomes compared with G1 tumors (both p < 0.001).

Receipt of systemic chemotherapy was associated with increased mortality (HR 1.71, 95% CI 1.36-2.17, p < 0.001). By contrast, cytoreductive surgery remained independently associated with improved survival, with a 20% reduction in mortality risk (HR 0.80, 95% CI 0.68-0.95, p = 0.011). Neither metastatic pattern (hepatic-only vs. extrahepatic) nor facility type/volume was independently associated with survival. Results were directionally consistent in the sensitivity analysis restricted to G1/G2 tumors, with age, comorbidity burden, surgical margins, higher tumor grade, receipt of systemic chemotherapy, and absence of CRS remaining significant predictors.

## Discussion

Management of metastatic GEP-NENs remains a subject of ongoing debate, largely due to the absence of prospective, randomized trials. Prior retrospective studies and single-institution experiences have reported symptomatic and survival benefits from surgical cytoreduction of liver metastases (9–15). Such trials included heterogeneous patient populations, varied surgical approaches, different surgical thresholds, and significant selection bias. Importantly, none directly compared surgical cytoreduction with systemic therapy, leaving the relative benefit of these approaches uncertain. Our study sought to address this gap by evaluating outcomes between CRS, systemic therapy, or a combination of the two using a large national cohort. To our knowledge, this represents the largest NCDB analysis to compare these treatment strategies in metastatic GEP-NENs.

In this study, we observed a significant overall survival benefit in patients with metastatic GEP-NENs who received surgical cytoreduction alone or in combination with systemic chemotherapy, compared with those who received medical therapy alone. The benefit was observed across all histological grades, with significantly higher OS in G1-G2 patients compared with G3 well-differentiated tumors. This aligns with previous data demonstrating that tumor grade is an important prognostic factor in metastatic GEP-NENs (18). Studies by Bertani et al. and Scott et al. similarly documented improved outcomes for patients with lower-grade disease undergoing extensive cytoreduction (15, 19).

The survival benefit in our study was independent of primary site, with both midgut and pancreatic NENs showing higher median OS with surgical cytoreduction. These findings were similar to those of Sarmeinto et al., who reported no significant difference in either 5-year survival rate or median OS between patients with small bowel and pancreatic NENs who underwent surgical cytoreduction (9). Subsequent studies by Maxwell et al. and Scott et al. reported comparable progression-free and overall survival benefits across primary sites among those who achieved substantial debulking (14, 15).

We also observed better outcomes among patients treated at academic centers than at community hospitals. While causation cannot be inferred, several factors may contribute to this difference, including the availability of specialized surgeons performing more complex, high-risk surgeries and the multidisciplinary approach to treating NENs at these academic institutions.

Although we adjusted for key clinical variables, this analysis has important limitations. Its retrospective design introduces selection bias, and the NCDB lacks granular details on chemotherapy regimens, liver-directed surgical techniques, and other systemic treatments. Histologic classifications of neuroendocrine neoplasms are updated in the NCDB only for patients diagnosed after 2018. Therefore, we used ICD-O-3 classifications for cases diagnosed before 2018 and a composite variable to ensure consistency across earlier cases. In our analysis, G3 tumors refer specifically to G3 well-differentiated NETs, as poorly differentiated neuroendocrine carcinomas (NECs) were excluded using ICD-O-3 histology codes. Because the NCDB does not reliably differentiate G3 NET from G3 NEC before 2018, some misclassification is possible. Additionally, the database cannot identify the use of peptide receptor radionuclide therapy (PRRT), approved in 2018 but not widely implemented until after 2020. Future studies with more granular treatment data and contemporary cohorts will be better positioned to evaluate the impact of PRRT and modern systemic therapies on clinical outcomes.

## Conclusion

The value of surgical cytoreduction for liver metastases in patients with metastatic GEP-NENs is confirmed. The survival benefit of surgical cytoreduction compared to systemic therapy alone was demonstrated, regardless of primary tumor site or histologic grade. In the absence of prospective randomized trials, the benefits and risks of surgical cytoreduction should be discussed with GEP-NEN patients who have liver metastases and are qualified for surgery. Additionally, choosing academic centers with high expertise in NENs may be associated with better outcomes, given the complexity of these surgeries. Further data is needed to validate these results in future prospective trials.

## Data Availability

Publicly available datasets were analyzed in this study. This data can be found here: NCDB is available at https://www.facs.org/quality-programs/cancer-programs/national-cancer-database/.
